# Interleukin-21 plays a critical role in the pathogenesis and severity of type I autoimmune hepatitis

**DOI:** 10.1186/s40064-016-2512-y

**Published:** 2016-06-18

**Authors:** Kazumichi Abe, Atsushi Takahashi, Hiromichi Imaizumi, Manabu Hayashi, Ken Okai, Yukiko Kanno, Hiroshi Watanabe, Hiromasa Ohira

**Affiliations:** Department of Gastroenterology and Rheumatology, Fukushima Medical University School of Medicine, 1 Hikarigaoka, Fukushima City, Fukushima 960-1295 Japan

**Keywords:** Autoimmune hepatitis, Interleukin-21, Follicular helper T cells, Severity

## Abstract

**Background and aims:**

Recently, the number of follicular helper T (Tfh) cells expressing interleukin (IL)-21 was found to increase in peripheral blood of human and murine models of autoimmune hepatitis (AIH). IL-21, the most recently discovered member of the type-I cytokine family, exerts various effects on the immune system, including B cell activation, plasma cell differentiation, and immunoglobulin production. We aimed to assess the relationship of serum IL-21 levels in patients with type I AIH with clinical and laboratory parameters and histology.

**Methods:**

Ninety-two Japanese patients with liver disease (22 AIH, 20 primary biliary cholangitis, 19 drug-induced liver injury, 8 acute hepatitis B, 8 chronic hepatitis C, 10 non-alcoholic steatohepatitis, 5 viral hepatitis) and 10 healthy volunteers were recruited. Serum IL-21 levels were detected by enzyme-linked immunosorbent assay. Real-time polymerase chain reaction measured mRNA levels of Bcl-6, IL-21, and CXCR5 (Tfh-related factors) in peripheral mononuclear cells.

**Results:**

Mean age at diagnosis of AIH was 58.6 years, male-to-female ratio was 4:18, 18.2 % of participants had cirrhosis, and 22.7 % had severe disease. IL-21 levels were significantly increased in the serum of patients with AIH compared to those with other liver diseases and controls (*p* < 0.0001). Particularly, serum IL-21 levels were significantly increased in severe AIH cases compared to non-severe cases (*p* < 0.05). Serum IL-21 levels correlated positively with total serum bilirubin levels (r = 0.46, *p* < 0.05), grading of necroinflammatory activity (r = 0.68, *p* < 0.005) and negatively with serum albumin levels in patients with AIH (r = −0.49, *p* < 0.05). In patients with biochemical remission of AIH, serum IL-21 levels remained elevated and correlated positively with serum IgG levels (r = 0.84, *p* < 0.01). Expression of Tfh-related factors, such as Bcl-6 and IL-21, in peripheral blood mononuclear cells of patients with AIH was significantly higher than that in healthy volunteers.

**Conclusions:**

IL-21 may play an important role in the pathogenesis and severity of AIH, and may present a promising target for AIH therapy.

**Electronic supplementary material:**

The online version of this article (doi:10.1186/s40064-016-2512-y) contains supplementary material, which is available to authorized users.

## Background

Autoimmune hepatitis (AIH) manifests as chronic liver inflammation of an unknown cause. It generally affects young to middle-aged women, and is associated with the presence of autoantibodies and hypergammaglobulinemia (Krawitt 1996). The histological features of interface hepatitis, i.e., the infiltration of lymphocytes, plasma cells, and macrophages, suggest the involvement of an aggressive cellular immune response in the pathogenesis of AIH (Vergani and Mieli-Vergani 2007). Human leukocyte antigen DR status affects the clinical features of patients with type 1 AIH. In Japanese patients, DR4 is dominantly associated with the disease. AIH is also associated with predominating Th1 responses and decreased function and number of regulatory T cells (Tregs) (Longhi et al. 2004, 2006).

Interleukin (IL)-21 is a member of the type I cyto-kine family (Zeng et al. 2007). The mature form of human IL-21 consists of 131 amino acids. The cytokine is produced by activated natural killer (NK) T cells and multiple CD4+ T cell subsets, including effector memory and central memory CD4+ T cells and differentiated T helper cell subsets polarized towards Th17 and T follicular helper (Tfh) phenotypes (Liu et al. 2009; Parrish-Novak et al. 2002; Wang et al. 2011). IL-21 alone is capable of directly inducing both B lymphocyte induced maturation protein-1 (Blimp-1), which is required for plasma cell differentiation, and the transcription factor B cell lymphoma 6 (Bcl-6), which is required for germinal center reactions (Ettinger et al. 2008). Overexpression of IL-21 in mice results in hypergammaglobulinemia and autoantibody production (Ozaki et al. 2004).

Differentiated Tfh cells express IL-21, IL-21 receptor (R), inducible costimulator (ICOS), CXC chemokine receptor (CXCR) 5, and programmed cell death protein-1 (PD-1). IL-21 and ICOS are indispensable for Tfh cell generation and the helper function of B cells. Moreover, IL-21 can modulate the activity of CD8+ T cells and other immune and non-immune cells in vivo. Since previous observations suggest that IL-21 and IL-21R may be associated with immunoglobulin production, autoantibody production, and B lymphocyte hyperactivity (Ozaki et al. 2002; Young et al. 2007), IL-21 may be involved in the pathogenesis of autoimmune diseases. Elevated amounts of IL-21 have subsequently been reported in many autoimmune diseases, including type 1 diabetes (King et al. 2004; McGuire et al. 2009), rheumatoid arthritis (Young et al. 2007; Sglunda et al. 2014), systemic lupus erythematosus (Ozaki et al. 2004; Bubier et al. 2009), Sjogren’s syndrome (Kang et al. 2011), inflammatory bowel diseases (Monteleone et al. 2005; Fina et al. 2008), and primary biliary cholangitis (PBC) (Wang et al. 2015).

In a mouse model of spontaneous fatal AIH, splenic CD4+ T cells were localized in B cell follicles with huge germinal centers, expressed Bcl-6 inducible ICOS, IL-21, and IL-21R, and were of the Tfh cell phenotype. Blocking antibodies to ICOS or IL-21 suppressed Tfh cell generation and the induction of AIH in this model. In addition, IL-21 produced by Tfh cells drove CD8+ T cell activation. Splenic Tfh cells and CD8+ T cells were found to express C–C chemokine receptor 6 (CCR6), and C–C chemokine ligand 20 (CCL20) was elevated in the liver. Finally, administration of an anti-CCL20 antibody suppressed the migration of these T cells to the liver and induction of AIH (Aoki et al. 2011). In another study using the same mouse model, IL-18 and the CXCR3/CXC chemokine ligand 9 (CXCL9) axis were demonstrated to be critical for T cell differentiation and migration (Ikeda et al. 2014).

A few studies have linked serum IL-21 levels to clinical outcomes in patients with AIH. For example, Ma et al. reported that the concentrations of serum IL-21 in patients with new onset AIH were correlated positively with serum immunoglobulin G (IgG), IgA, and IgM (Ma et al. 2014). However, the roles of IL-21 in AIH remain poorly understood. This study aimed to assess the role of serum IL-21 in the pathogenesis and severity of type I AIH in Japanese patients.

## Methods

### Patients

Subjects were 22 patients with AIH, 20 with PBC, 19 with drug-induced liver injury (DILI), 8 with acute hepatitis B, 8 with chronic hepatitis C (CHC), 10 with non-alcoholic steatohepatitis (NASH), and 5 with viral hepatitis, who received a diagnosis at Fukushima Medical University Hospital between 1997 and 2014, and 10 healthy controls. As healthy controls, normal serum was taken from staff members of our department. The diagnosis of AIH was based on the revised and simplified International Autoimmune Hepatitis Group (IAIHG) scoring system (Johnson and McFarlane 1993; Alvarez et al. 1999; Hennes et al. 2008). Patients with other causes of chronic liver disease, particularly alcohol abuse, chronic hepatitis B, or CHC, were excluded from the AIH group. Patients were diagnosed as having PBC features if they met at least two of the following three criteria: (1) chronic elevation of cholestatic liver enzymes alkaline phosphatase (ALP) and gamma-glutamyltranspeptidase (GTP) for at least 6 months; (2) presence of serum anti-mitochondrial antibody (AMA), detected by either indirect immunofluorescence or ELISA using commercially available kits; and (3) typical histological findings from biopsied liver specimens (Lindor et al. 2009). The diagnosis of DILI was made by hepatologists at our hospital with due consideration of alternative causes and reactions to drugs. In addition, confirmation of a NASH diagnosis was made by liver biopsy in the absence of other liver diseases. Viral hepatitis included that resulting from infection with 3 Epstein–Barr viruses, 1 cytomegalovirus, and 1 hepatitis A virus.

Assessed data that were extracted retrospectively from patients’ medical charts included patient background (age, sex, onset type of disease, IAIHG score), clinical parameters at presentation [AST, ALT, ALP, TB, Alb, PLT, prothrombin time (PT), IgG, antinuclear antibodies (ANA)] and at remission (ALT, ALP, TB, IgG), presence of relapse, cirrhosis, severity of disease, and therapeutic methods. Acute AIH was defined by the presence of acute onset of symptoms (e.g., jaundice and/or fatigue and/or anorexia) in conjunction with bilirubin >5 mg/dl and/or serum ALT levels higher than tenfold the upper normal limit. Relapse of AIH was defined as an increase in serum transaminases to greater than twice the upper normal limit (ALT levels >90 U/l). Maintenance of serum transaminase levels of less than twice the upper limit of normal during follow-up constituted a sustained remission. Liver cirrhosis was comprehensively diagnosed by liver biopsy findings and/or clinical parameters (including a PLT count of 10 × 10^4^/μl or less and hyaluronic acid levels of 130 ng/ml or higher), presence of complications, and imaging findings (image pattern in abdominal ultrasound and CT findings). Disease severity was assessed with the diagnosis and treatment guide for AIH in Japan (Onji et al. 2014). Severe cases were defined as those that fulfilled at least one of the following: (1) clinical signs: (a) hepatic encephalopathy, (b) reduction or disappearance of hepatic dullness, (2) clinical laboratory tests: (a) AST/ALT > 200 U/l, (b) bilirubin > 5 mg/dl, (c) PT < 60 %, and (3) imaging tests: (a) hepatic atrophy, (b) heterogenous liver parenchyma pattern. Eight (36 %) of 22 patients with AIH who had achieved biochemical remission (defined as normal serum ALT and IgG levels) were assessed after 60.2 months (range 16–167) of treatment. Serum cytokine and chemokine levels (IL-21, IL-18, CCL20, CCR6, CXCL9, CXCR3) were detected by using serum samples. The expression levels of Bcl-6, IL-21, and CXCR5 were measured by using mRNA in peripheral blood mononuclear cells (PBMCs). These samples were obtained at the time of diagnosis or remission and stored unthawed in several tubes at −20 °C until testing.

### Ethics statement

This study was approved for the use of an opt-out consent by the ethics committee of Fukushima Medical University School of Medicine. A website with additional information and including an opt-out consent was set up for the study. All patients agreed to serum testing and written informed consent was obtained. Retrospective testing of 92 blood samples from patients and volunteers was performed as part of the approved protocol and data were analyzed anonymously according to the institutional and national ethics rules.

### Enzyme-linked immunosorbent assays (ELISA)

ELISA kits were used to determine the levels of serum IL-21 (Creative Diagnostics, NY, USA), IL-18 (MBL, Nagoya, Japan), CCL20, CXCL9 (R&D, Minneapolis, USA), CCR6 (CUSABIO, Hubei, China), and CXCR3 (Cloud-clone, Houston, TX, USA), according to the manufacturer’s instructions.

### Quantitative RT-PCR

Peripheral blood was drawn from patients with AIH and volunteers into a tube containing EDTA-2Na and centrifuged to separate PBMCs. RNA was reverse transcribed to single stranded cDNA using the Random Primer, dNTP Mixture (Takara Shuzo Co., Ltd., Shiga, Japan) and RNasin^®^ Ribonuclease Inhibitor (Promega, Madison, WI, USA), according to the manufacturer’s protocol. cDNA was used for quantitative analysis by PCR. Quantitative real time PCR (qPCR) was performed with a LightCycler 2.0 System (Roche Applied Science, Germany) using LightCycler DNA Master SYBR Green I (Roche Applied Science). PCR mixtures contained 0.5 μM sense and antisense primers. Samples were denatured at 95 °C for 10 min, followed by 45 cycles of annealing and extension at 95 °C for 15 s, 60 °C for 5 s, and 72 °C for 10 s. Melting curves were obtained at the end of amplification by cooling the samples to 65 °C for 15 s, followed by further cooling to 40 °C for 30 s. Data were analyzed by the standard curve method for relative quantification using LightCycler analysis software. For qPCR, data were normalized against GAPDH.

**Table:** 

RT-PCR primer		Sequence
GAPDH	F	5′-GGTGAAGGTCGGAGTCAACGG-3′
	R	5′-TGAAGGGGTCATTGATGGCAACA-3′
CXCR5	F	5′-GGTCTTCATCTTGCCCTTTG-3′
	R	5′-ATGCGTTTCTGCTTGGTTCT-3′
Bcl-6	F	5′-GAAGCCCTACAAATGCGAAA-3′
	R	5′-TGACGGAAATGCAGGTTACA-3′
IL-21	F	5′-CCACAAATCAAGCTCCCAAG-3′
	R	5′-CAGGGACCAAGTCATTCACA-3′

### Histological evaluation

AIH patients underwent liver biopsy with ultrasound guidance. Three patients were excluded from pathological analysis: one because of ascites, and the other because of cirrhosis. Thus, 19 patients were included for further assessment. The liver sections stained by hematoxylin and eosin. Slides were coded and read by two pathologists who were unaware of the patient’s identity and history. Histological evaluation was performed according to the classification of Scheuer (1991) and Desmet et al. (1994). Grading of necroinflammatory activity (G) and staging of fibrosis (S) ranged from G 0–4 and S 0–4, respectively.

### Statistical analyses

Results are expressed as mean ± SD. Differences were compared using the Mann–Whitney U-test and Wilcoxon matched-pairs signed-rank test. Correlations between variables were assessed using Spearman’s rank correlation coefficient. To find the optimal cut-off level of TB, IL-21 and CCL20 that would distinguish between severe and non-severe necroinflammatory activity, receiver operating characteristic (ROC) curves were used. Cut-off levels for parameters were set as the points closest to 100 % sensitivity and specificity. Multivariate logistic regression analysis was performed for univariate or multivariate analysis on factors related to the grading of necroinflammatory activity. All statistical analyses were performed using Prism 6.0 software (GraphPad Software, Inc) and Excel Statistics (2011, Esumi, Co. Ltd., Tokyo, Japan). *p* < 0.05 was considered significant.

## Results

### Patients

Table 1 shows the characteristics of patients with AIH. In patients with AIH, male to female ratio was 4:18, mean age at diagnosis was 58.6 years. Acute and chronic AIH accounted for 50.0 % of patients each. Revised IAIHG scores averaged 15.1 points before treatment, and simplified IAIHG scores averaged 6.2 points. Laboratory data at diagnosis were as follows: total bilirubin (TB), 7.3 ± 9.7 mg/dl; ALT, 537.8 ± 732.1 U/l; alkaline phosphatase (ALP), 440.8 ± 240.6 U/l; Alb, 3.3 ± 0.7 g/dl; and PT, 72.9 ± 22.9 %. Serum IgG at onset was 2604.1 ± 1021.7 mg/dl. The median titer of antinuclear antibodies (ANA) was 1:160. Five patients (22.7 %) had severe disease. The grading of necroinflammatory activity was G0 (0/19, 0 %), G1 (2/19, 10.5 %), G2 (9/19, 47.4 %), G3 (7/19, 36.8 %), G4 (1/19, 5.3 %). The staging of fibrosis was F0 (2/19, 10.5 %), F1 (4/19, 21.1 %), F2 (5/19, 26.3 %), F3 (6/19, 31.6 %), F4 (2/19, 10.5 %). With respect to therapy, most patients (72.7 %) were treated with prednisolone (PSL) alone; three (13.6 %) were treated with ursodeoxycholic acid (UDCA) alone, and three (13.6 %) were treated with azathioprine (AZA) in combination with PSL. When clinical parameters at remission were compared with those at onset, ALT (13.3 ± 6.9 U/l), ALP (239.8 ± 81.6 U/l), TB (0.9 ± 0.4 mg/dl), and IgG (1236.5 ± 249.6 mg/dl) were significantly lower and were within the normal range. Characteristics of patients with other liver diseases are summarized in Additional file 1: Table 1.

**Table 1:** Characteristics of patients with autoimmune hepatitis

Age (year)	58.6 ± 11.7
Gender (male/female)	4/18
Onset type (acute/chronic)	11/11
AIH score (revised)	15.1 ± 4.0
AIH score (simplified)	6.2 ± 1.3
AST (U/l)	613.4 ± 831.1
ALT (U/l) onset/remission	537.8 ± 732.1/13.3 ± 6.9
ALP (U/l) onset/remission	440.8 ± 240.6/239.8 ± 81.6
Alb (g/dl)	3.3 ± 0.7
TB (mg/dl) onset/remission	7.3 ± 9.7/0.9 ± 0.4
PT (%)	72.9 ± 22.9
PLT (×10^4^/μl)	15.0 ± 5.1
IgG (mg/dl) onset/remission	2604.1 ± 1021.7/1236.5 ± 249.6
ANA (median)	160
Relapse n (%)	7 (31.8)
Grading of necroinflammatory activity 0/1/2/3/4 (n)	0/2/9/7/1
Staging of fibrosis 0/1/2/3/4 (n)	2/4/5/6/2
UDCA monotherapy n (%)	3 (13.6)
PSL monotherapy n (%)	16 (72.7)
PSL + AZA n (%)	3 (13.6)

*AIH* autoimmune hepatitis, *AST* aspartate aminotransferase (normal: 13–33 U/l), *ALT* alanine aminotransferase (normal: 6–27 U/l), *ALP* alkaline phosphatase (normal: 115–359 U/l), *IgG* immunoglobulin G (normal: 870–1700 mg/dl), *ANA* antinuclear antibody (normal: 0–39×), *UDCA* ursodeoxycholic acid, *PSL* prednisolone, *AZA* azathioprine

### Patients with AIH have higher serum IL-21 levels than those with other liver diseases and healthy controls

**Fig. 1:**
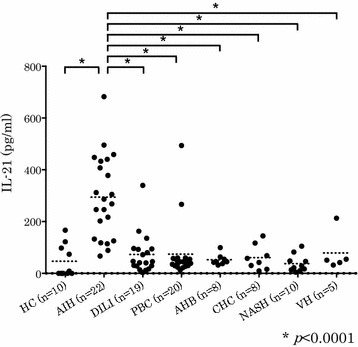
Patients with AIH have higher serum IL-21 levels than those with other liver diseases and healthy controls. *p* values were calculated with the Mann–Whitney test (*p < 0.0001)

The mean titer of IL-21 by ELISA in serum samples from patients with AIH (294.3 pg/ml) was significantly higher than in those with DILI (72.8 pg/ml, *p* < 0.0001), PBC (74.1 pg/ml, *p* < 0.0001), acute hepatitis B (52.6 pg/ml, *p* < 0.0001), CHC (60.8 pg/ml, *p* < 0.0001), NASH (37.9 pg/ml, *p* < 0.0001), and viral hepatitis (78.6 pg/ml, *p* < 0.0001), and in sera from healthy volunteers (46.9 pg/ml, *p* < 0.0001) (Fig. 1).

### Expression of Tfh-related factors in peripheral blood mononuclear cells of patients with AIH

**Fig. 2:**
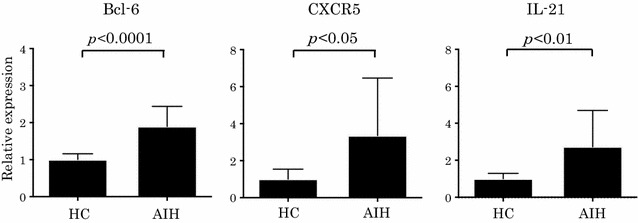
Expression of Tfh-related factors in peripheral blood mononuclear cells of patients with AIH. *p* values were calculated with the Mann–Whitney test

As shown in Fig. 2, there were significant differences in the mRNA expression of follicular helper T (Tfh)-related factors in PBMCs between healthy controls and patients with AIH. Expression of Tfh-related factors, such as Bcl-6, IL-21, and CXCR5, in patients with AIH was significantly higher compared to that in healthy controls.

### Comparison between patients with severe and non-severe AIH at presentation

As shown in Table 2, patients with AIH were also assessed by severity of disease. When patient background and clinical parameters at diagnosis were compared between patients with severe and non-severe (mild and moderate) AIH, Alb (2.7 vs. 3.5 g/dl, *p* < 0.05) and PT (46.4 vs. 81.3 %, *p* < 0.005) were significantly lower in the severe AIH group, and serum IL-21 (396.7 vs. 264.7 pg/ml, *p* < 0.05) and IL-18 (1776.6 vs. 808.6 pg/dl, *p* < 0.05) were significantly higher in the severe AIH group.

**Table 2:** Comparison of clinical and laboratory parameters between severe and non-severe autoimmune hepatitis

	Non-severe (n = 17)			
	Mild (n = 4)	Moderate (n = 13)	Severe (n = 5)	*p*
ALT (U/l)	96 ± 66	599 ± 858	729 ± 590	0.2488
Alb (g/dl)	4.0 ± 0.2	3.4 ± 0.6	2.7 ± 0.8	<0.05*
TB (mg/dl)	0.9 ± 0.3	7.5 ± 10.7	12.0 ± 8.9	0.1138
PT (%)	95.5 ± 2.8	77.9 ± 19.4	46.4 ± 12.2	<0.005*
PLT (×10^4^/μl)	20.9 ± 5.4	14.6 ± 4.4	11.5 ± 2.9	0.1117
IgG (mg/dl)	2834 ± 725	2415 ± 1112	2910 ± 1042	0.4455
IL-21 (pg/ml)	244.5 ± 146.6	270.3 ± 173.4	396.7 ± 99.9	<0.05*
IL-18 (pg/ml)	366.2 ± 213.2	944.8 ± 846.9	1776.6 ± 891.2	<0.05*
CCL20 (pg/ml)	58.4 ± 45.9	215.3 ± 316.9	255.2 ± 247.7	0.2488
CCR6 (pg/ml)	26.2 ± 9.2	147.8 ± 133.7	163.9 ± 71.2	0.1196
CXCL9 (pg/ml)	1206.7 ± 1370.2	1419.9 ± 1004.9	1953.5 ± 903.1	0.2488
CXCR3 (ng/ml)	43.7 ± 61.0	54.4 ± 34.7	86.8 ± 47.5	0.1012

*AST* aspartate aminotransferase, *ALT* alanine aminotransferase, *ALP* alkaline phosphatase, *IgG* immunoglobulin G, *IL-21* interleukin 21, *IL-18* interleukin 18, *CCL20* C–C chemokine ligand 20, *CCR6* C–C chemokine receptor 6, *CXCL9* CXC chemokine ligand 9, *CXCR3* CXC chemokine receptor 3

*p* values were calculated with the Mann–Whitney U test (*statistically significant)

### Relationship between serum IL-21 and clinical presentation

Serum IL-21 levels were significantly and positively correlated with TB, CCL20, CXCL9, and CXCR3 levels, and negatively correlated with Alb levels (Table 3).

**Table 3:** Relationship between serum IL-21 levels and clinical presentation in patients with autoimmune hepatitis

Variable	r	*p*
AST (U/l)	0.2716	0.2215
ALT (U/l)	0.2433	0.2753
ALP (U/l)	0.2366	0.2890
Alb (g/dl)	−0.4951	<0.05*
TB (mg/dl)	0.4641	<0.05*
PT (%)	−0.3587	0.1103
PLT (×10^4^/μl)	−0.2981	0.1778
IgG (mg/dl)	0.2928	0.1860
ANA (median)	0.0335	0.8853
IL-18 (pg/ml)	0.1158	0.6079
CCL20 (pg/ml)	0.5987	<0.005*
CCR6 (pg/ml)	0.4151	0.0547
CXCL9 (pg/ml)	0.4999	<0.05*
CXCR3 (ng/ml)	0.5016	<0.05*

*AST* aspartate aminotransferase, *ALT* alanine aminotransferase, *ALP* alkaline phosphatase, *IgG* immunoglobulin G, *ANA* antinuclear antibody, *IL-18* interleukin-18, *CCL20* C–C chemokine ligand 20, *CCR6* C–C chemokine receptor 6, *CXCL9* CXC chemokine ligand 9, *CXCR3* CXC chemokine receptor 3

*p* values were calculated with the Spearman rank correlation test (*statistically significant)

### Relationship between serum IL-21 and histology

**Fig. 3:**
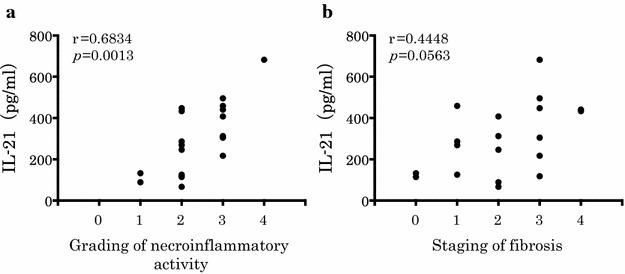
Relationship between serum IL-21 and histology. Serum IL-21 levels according to the degree of necroinflammatory activity and liver fibrosis.** a** Grading of necroinflammatory activity, **b** staging of fibrosis. Although serum IL-21 levels did not correlate with staging of fibrosis (r = 0.45, * p* = 0.056), they were significantly positively correlated with grading of necroinflammatory activity (r = 0.68, *p* < 0.005).* p* values were calculated with Spearman rank correlation test

Spearman’s rank coefficient analysis showed in Fig. 3. Although serum IL-21 levels did not correlate with staging of fibrosis (r = 0.45, *p* = 0.056), they were significantly positively correlated with grading of necroinflammatory activity (r = 0.68, *p* < 0.005). As shown in Table 4, patients with AIH were also assessed by progression of necroinflammatory activity. When clinical and laboratory parameters at diagnosis were compared between severe necroinflammatory activity (G 3–4) and non-severe (G 0–2) in liver histology, TB (3.2 vs. 11.0 mg/dl, *p* < 0.05), IL-21 (211.7 vs. 414.9 pg/ml, *p* < 0.01) and CCL20 (55.4 vs. 329.3 pg/ml, *p* < 0.01) were significantly higher in the severe group. The results of multivariate logistic regression analysis using the 3 factors, significantly associated with severe necroinflammatory activity, were shown in Table 5. To find the optimal cut-off level of TB, IL-21 and CCL20 that would distinguish between severe and non-severe necroinflammatory activity, ROC curves were used. It was set at more than 5.2 mg/dl in TB (*p* < 0.05) at more than 296 pg/ml in serum IL-21 (*p* < 0.01) and at more than 71 pg/ml in serum CCL20 (*p* < 0.01). With the significant factors extracted by univariate analysis, multivariate analysis was performed, and IL-21 of more than 296 pg/ml was independent factors.

**Table 4:** Comparison of clinical and laboratory parameters between severe and non-severe grading of necroinflammatory activity in liver histology

	Grading of necroinflammatory activity: G0–2 (n = 11)	Grading of necroinflammatory activity: G3–4 (n = 8)	*p*
ALT (U/l)	420 ± 426	818 ± 1088	0.3484
Alb (g/dl)	3.5 ± 0.8	3.0 ± 0.8	0.0860
TB (mg/dl)	3.2 ± 6.4	11.0 ± 9.6	<0.05*
PT (%)	68.7 ± 28.4	74.1 ± 18.6	0.9150
PLT (×10^4^/μl)	16.5 ± 6.2	13.1 ± 4.2	0.2014
IgG (mg/dl)	2307 ± 1131	2836 ± 548	0.1767
IL-21 (pg/ml)	211.7 ± 135.3	414.9 ± 142.7	<0.01*
IL-18 (pg/ml)	903.8 ± 667.4	1069.2 ± 891.9	0.8910
CCL20 (pg/ml)	55.4 ± 62.5	329.3 ± 302.8	<0.01*
CCR6 (pg/ml)	113.3 ± 112.6	142.5 ± 99.1	0.4381
CXCL9 (pg/ml)	1356.8 ± 1036.9	2020.8 ± 963.6	0.1084
CXCR3 (ng/ml)	51.7 ± 43.6	82.9 ± 41.6	0.1084

*AST* aspartate aminotransferase, *ALT* alanine aminotransferase, *ALP* alkaline phosphatase, *IgG* immunoglobulin G, *IL-21* interleukin 21, *IL-18* interleukin 18, *CCL20* C–C chemokine ligand 20, *CCR6* C–C chemokine receptor 6, *CXCL9* CXC chemokine ligand 9, *CXCR3* CXC chemokine receptor 3

*p* values were calculated with the Mann–Whitney U test (*statistically significant)

**Table 5:** Factors related to grading of necroinflammatory activity (G3–G4 vs. G0–G2): univariate analysis and multivariate analysis

Factors	Univariate analysis			Multivariate analysis		
	OR	95 % CI	*p*	OR	95 % CI	*p*
TB (mg/dl)						
5.2>	1 (Ref)			1 (Ref)		
5.2<	7.50	0.91–61.93	0.061	4.73	0.12–178.76	0.402
IL-21 (pg/ml)						
296>	1 (Ref)			1 (Ref)		
296<	31.50	2.53–392.92	<0.01*	23.94	1.15–499.49	<0.05*
CCL20 (pg/ml)						
71>	1 (Ref)			1 (Ref)		
71<	12.25	1.35–111.01	<0.05*	1.23	0.03–43.26	0.910

*Ref* reference group, *IL-21* interleukin 21, *CCL20* C–C chemokine ligand 20

*p* values were calculated with multiple logistic regression analysis (*statistically significant)

### Relationship between serum IL-21 and IgG levels at onset and remission in patients with AIH

**Fig. 4:**
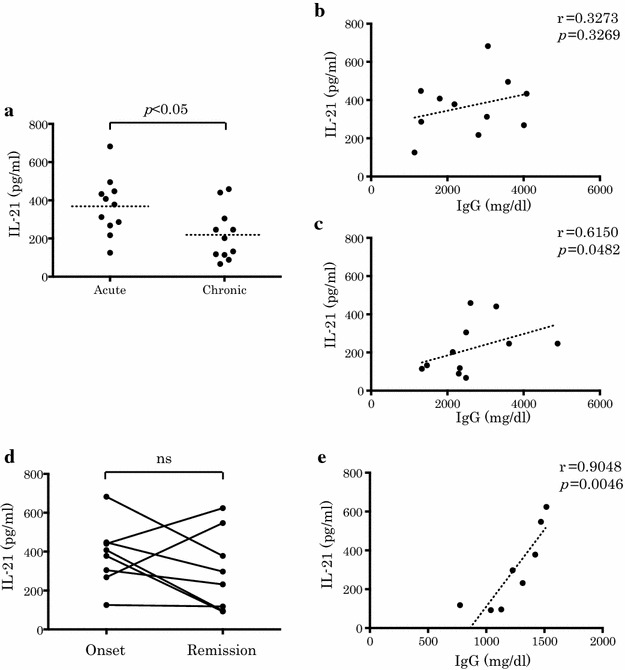
Relationship between serum IL-21 and IgG levels at onset and remission in patients with AIH. **a** Comparison between AIH patients at onset of acute (n = 11) or chronic (n = 11) presentation. **b** Relationship between serum IL-21 levels and serum IgG levels in patients with AIH at onset of acute AIH. **c** Relationship between serum IL-21 levels and serum IgG levels in patients with AIH at onset of chronic AIH. **d** Comparison between AIH patients at onset and remission (n = 8). **e** Relationship between serum IL-21 levels and serum IgG levels in patients with AIH at remission. *p* values were calculated with the Mann–Whitney test, Spearman rank correlation test, or Wilcoxon matched-pairs signed-rank test, *ns* not significant

The relationship between serum IL-21 and IgG levels in patients with AIH is shown in Fig. 4. Serum IL-21 levels were significantly higher in patients with acute AIH compared to those with chronic AIH (*p* < 0.05). Although serum IL-21 levels did not significantly correlate with serum IgG levels in patients with acute AIH, they were significantly and positively correlated with serum IgG levels in those with chronic AIH. Surprisingly, serum IL-21 levels did not significantly differ in patients with AIH at onset and remission. Serum IL-21 levels were significantly and positively correlated with serum IgG levels in patients with AIH at remission (*p* < 0.005).

## Discussion

The main finding of this study was that serum IL-21 levels were significantly increased in the serum of patients with AIH compared to those with other liver diseases and controls. In particular, serum IL-21 levels were significantly increased in patients with severe AIH compared to those with non-severe AIH. Moreover, serum IL-21 levels were significantly positively correlated with grading of necroinflammatory activity in liver histology.

In a previous study, serum IL-21 levels in patients with chronic hepatitis B (CHB) and hepatitis B-related acute-on-chronic liver failure were significantly increased compared to levels in healthy controls (Hu et al. 2011). In another study, serum IL-21 levels at treatment week 12 were significantly higher in CHB patients who achieved a complete response, compared to those who did not (Ma et al. 2012). In the present study, serum IL-21 levels were not increased in patients with acute hepatitis B.

In a recent report, patients with PBC were found not only to have increased Tfh cells, but also increased IL-21 levels and B cell activation, disease severity, and responsiveness to UDCA therapy (Wang et al. 2015). Our results showed that serum IL-21 levels in some patients with PBC were increased, and that the levels in patients with AIH were significantly higher than in those with DILI. DILI with features of autoimmunity represents an important category of hepatotoxicity due to medication exposure. In daily clinical practice, distinguishing DILI from acute AIH is often difficult (Suzuki et al. 2011; Fujiwara and Yokosuka 2012), and in this context, serum IL-21 could potentially be useful for differential diagnosis.

Several studies have demonstrated a correlation between the severity of autoimmune diseases and IL-21 levels (Choi et al. 2015; Szabo et al. 2013; He et al. 2012; Rasmussen et al. 2010). In the present study, serum IL-21 levels were significantly higher in the severe AIH group and positively correlated with serum TB, CCL20, CXCL9 and CXCR3 levels. In a mouse model of AIH, blocking IL-21 suppressed Tfh cell generation and the induction of AIH, and IL-21 produced by Tfh cells was shown to drive CD8+ T-cell activation (Aoki et al. 2011). Hepatic macrophages/Kupffer cells producing CXCL9 are critical for the migration of CXCR3-expressing T cells, and dendritic cell-derived IL-18 is important for the differentiation of Th1 cells and CD8+ effector T cells (Ikeda et al. 2014). These systems may serve as targets for treating patients with severe AIH. As demonstrated in the present study, serum IL-21 levels, with a cut-off of 296 pg/ml, may predict the progression of necroinflammatory activity in liver histology.

A recent nationwide survey suggested that serum IgG levels at the onset of AIH decreased compared with those in a previous study (Abe et al. 2011). In addition, patients with acute AIH occasionally present with lower serum IgG levels and/or the absence or low titers of serum autoantibodies (Onji 2011). In the present study, serum IL-21 levels were significantly higher in patients with acute AIH compared to those with chronic AIH. Although serum IL-21 levels were not significantly correlated with serum IgG levels in acute AIH, they were significantly and positively correlated with serum IgG levels in chronic AIH.

In patients with AIH in biochemical remission, serum IL-21 levels were still elevated, despite a reduction in serum IL-18, CCL20, CCR6, CXCL9, and CXCR3 levels (Additional file 2: Figure 1), and significantly and positively correlated with serum IgG levels. Biochemical remission is typically defined as the normalization of serum transaminases and IgG. The Mayo Clinic trial showed that histological remission lagged behind biochemical remission by several months and was achieved by only 60 % of patients after 2-year treatment with PSL ± AZA (Soloway et al. 1972). Another report showed that persistent histological activity, despite biochemical remission, is frequently observed in patients treated for AIH and is associated with lower rates of fibrosis regression and reduced long-term survival (Dhaliwal et al. 2015).

This study has several limitations. First, the expression of Tfh-related factors, such as Bcl-6, CXCR5 and IL-21, in peripheral blood mononuclear cells of patients with AIH was significantly increased compared to the expression in healthy volunteers. However, the expression of these factors in liver tissue was not investigated. Second, the sample population was relatively small. Finally, this study was retrospective in design, and thus our results will need to be confirmed in a prospective study.

## Conclusions

Our findings suggest that IL-21 may play an important role in the pathogenesis and severity of AIH. Further research on the systemic and localized effects of IL-21 in AIH will provide a basis for targeted therapy that could benefit this patient population.

## Additional files

10.1186/s40064-016-2512-y Characteristics of patients with other liver diseases.
10.1186/s40064-016-2512-y Comparison between AIH patients at onset and remission. Serum cytokine and chemokine (CCL20, CCR6, IL-18, CXCL9, CXCR3) levels were reduced in patients with AIH at the time of remission (n = 8). P values were calculated with Wilcoxon matched-pairs signed-rank test.

## Abbreviations

AST: aspartate aminotransferase
ALT: alanine aminotransferase
ALP: alkaline phosphatase
GTP: gamma-glutamyltranspeptidase
TB: total bilirubin
IgG: immunoglobulin G
ANA: antinuclear antibody
AMA: anti-mitochondrial antibody
PBMCs: peripheral blood mononuclear cells
ELISA: enzyme-linked immunosorbent assays
qPCR: quantitative real time PCR
IL-18: interleukin-18
CCL20: C–C chemokine ligand 20
CCR6: C–C chemokine receptor 6
CXCL9: CXC chemokine ligand 9
CXCR3: CXC chemokine receptor 3
AIH: autoimmune hepatitis
PBC: primary biliary cholangitis
DILI: drug-induced liver injury
CHC: chronic hepatitis C
CHB: chronic hepatitis B
NASH: non-alcoholic steatohepatitis
IAIHG: International Autoimmune Hepatitis Group
UDCA: ursodeoxycholic acid
PSL: prednisolone
AZA: azathioprine
Tregs: regulatory T cells
NK: natural killer
Tfh: T follicular helper
Blimp-1: B lymphocyte induced maturation protein-1
ICOS: inducible costimulator
PD-1: programmed cell death protein-1
ROC: receiver operating characteristic
