# Supplementary material for: Interleukin-21 plays a critical role in the pathogenesis and severity of type I autoimmune hepatitis
Source: Springerplus. 2016 Jun 18;5(1):777. doi: 10.1186/s40064-016-2512-y (PMC4912506; doi:10.1186/s40064-016-2512-y)
Supplement: Supplementary file 1 — 10.1186/s40064-016-2512-y Characteristics of patients with other liver diseases. [file 40064_2016_2512_MOESM1_ESM.pptx]

## Slide 1
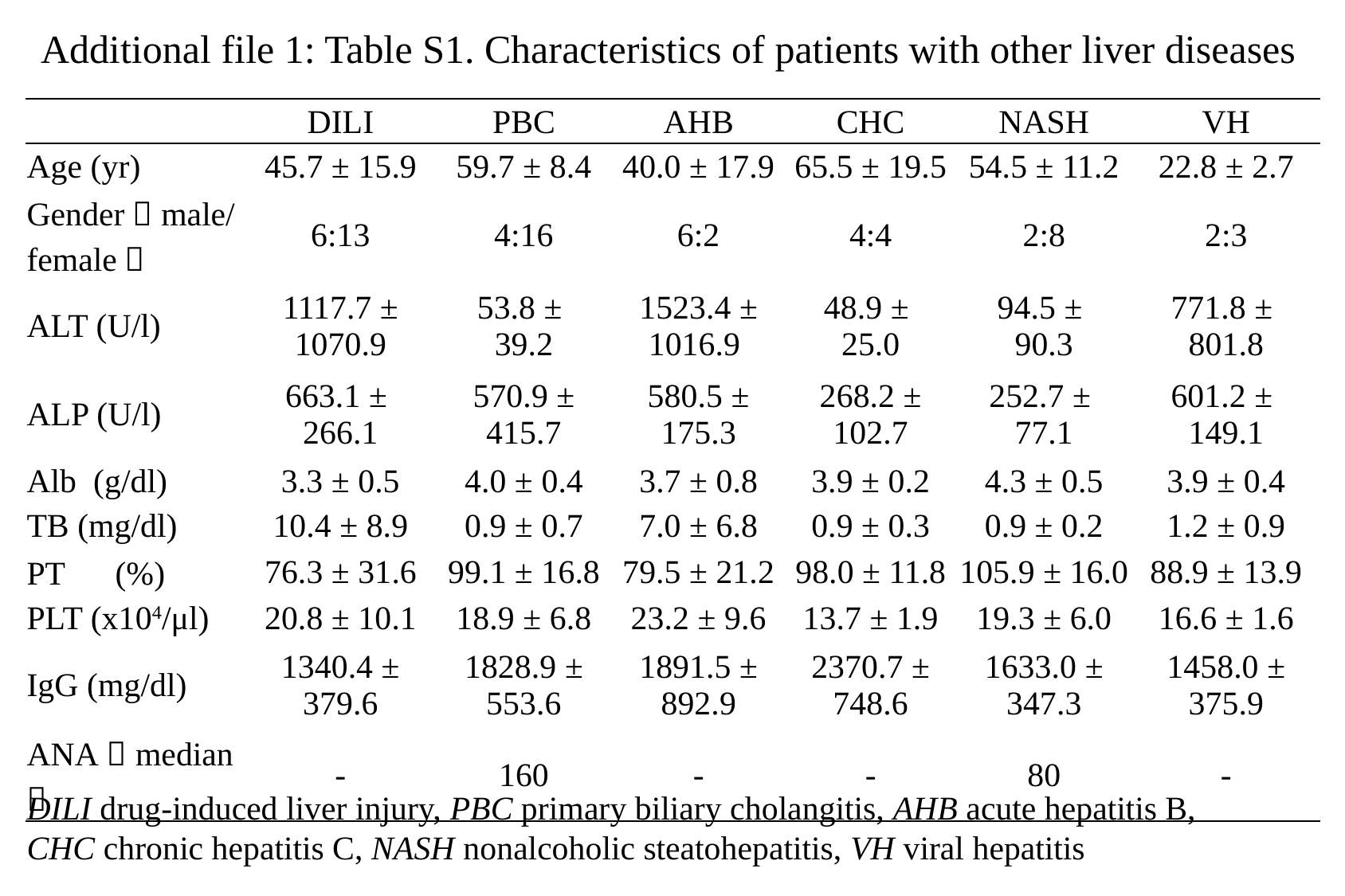

Additional file 1: Table S1. Characteristics of patients with other liver diseases
| | DILI | PBC | AHB | CHC | NASH | VH |
| --- | --- | --- | --- | --- | --- | --- |
| Age (yr) | 45.7 ± 15.9 | 59.7 ± 8.4 | 40.0 ± 17.9 | 65.5 ± 19.5 | 54.5 ± 11.2 | 22.8 ± 2.7 |
| Gender（male/female） | 6:13 | 4:16 | 6:2 | 4:4 | 2:8 | 2:3 |
| ALT (U/l) | 1117.7 ± 1070.9 | 53.8 ± 39.2 | 1523.4 ± 1016.9 | 48.9 ± 25.0 | 94.5 ± 90.3 | 771.8 ± 801.8 |
| ALP (U/l) | 663.1 ± 266.1 | 570.9 ± 415.7 | 580.5 ± 175.3 | 268.2 ± 102.7 | 252.7 ± 77.1 | 601.2 ± 149.1 |
| Alb (g/dl) | 3.3 ± 0.5 | 4.0 ± 0.4 | 3.7 ± 0.8 | 3.9 ± 0.2 | 4.3 ± 0.5 | 3.9 ± 0.4 |
| TB (mg/dl) | 10.4 ± 8.9 | 0.9 ± 0.7 | 7.0 ± 6.8 | 0.9 ± 0.3 | 0.9 ± 0.2 | 1.2 ± 0.9 |
| PT　(%) | 76.3 ± 31.6 | 99.1 ± 16.8 | 79.5 ± 21.2 | 98.0 ± 11.8 | 105.9 ± 16.0 | 88.9 ± 13.9 |
| PLT (x104/μl) | 20.8 ± 10.1 | 18.9 ± 6.8 | 23.2 ± 9.6 | 13.7 ± 1.9 | 19.3 ± 6.0 | 16.6 ± 1.6 |
| IgG (mg/dl) | 1340.4 ± 379.6 | 1828.9 ± 553.6 | 1891.5 ± 892.9 | 2370.7 ± 748.6 | 1633.0 ± 347.3 | 1458.0 ± 375.9 |
| ANA（median） | - | 160 | - | - | 80 | - |
DILI drug-induced liver injury, PBC primary biliary cholangitis, AHB acute hepatitis B,
CHC chronic hepatitis C, NASH nonalcoholic steatohepatitis, VH viral hepatitis
