# Supplementary material for: Interleukin-21 plays a critical role in the pathogenesis and severity of type I autoimmune hepatitis
Source: Springerplus. 2016 Jun 18;5(1):777. doi: 10.1186/s40064-016-2512-y (PMC4912506; doi:10.1186/s40064-016-2512-y)
Supplement: Supplementary file 2 — 10.1186/s40064-016-2512-y Comparison between AIH patients at onset and remission. Serum cytokine and chemokine (CCL20, CCR6, IL-18, CXCL9, CXCR3) levels were reduced in patients with AIH at the time of remission (n = 8). P values were calculated with Wilcoxon matched-pairs signed-rank test. [file 40064_2016_2512_MOESM2_ESM.pptx]

## Slide 1
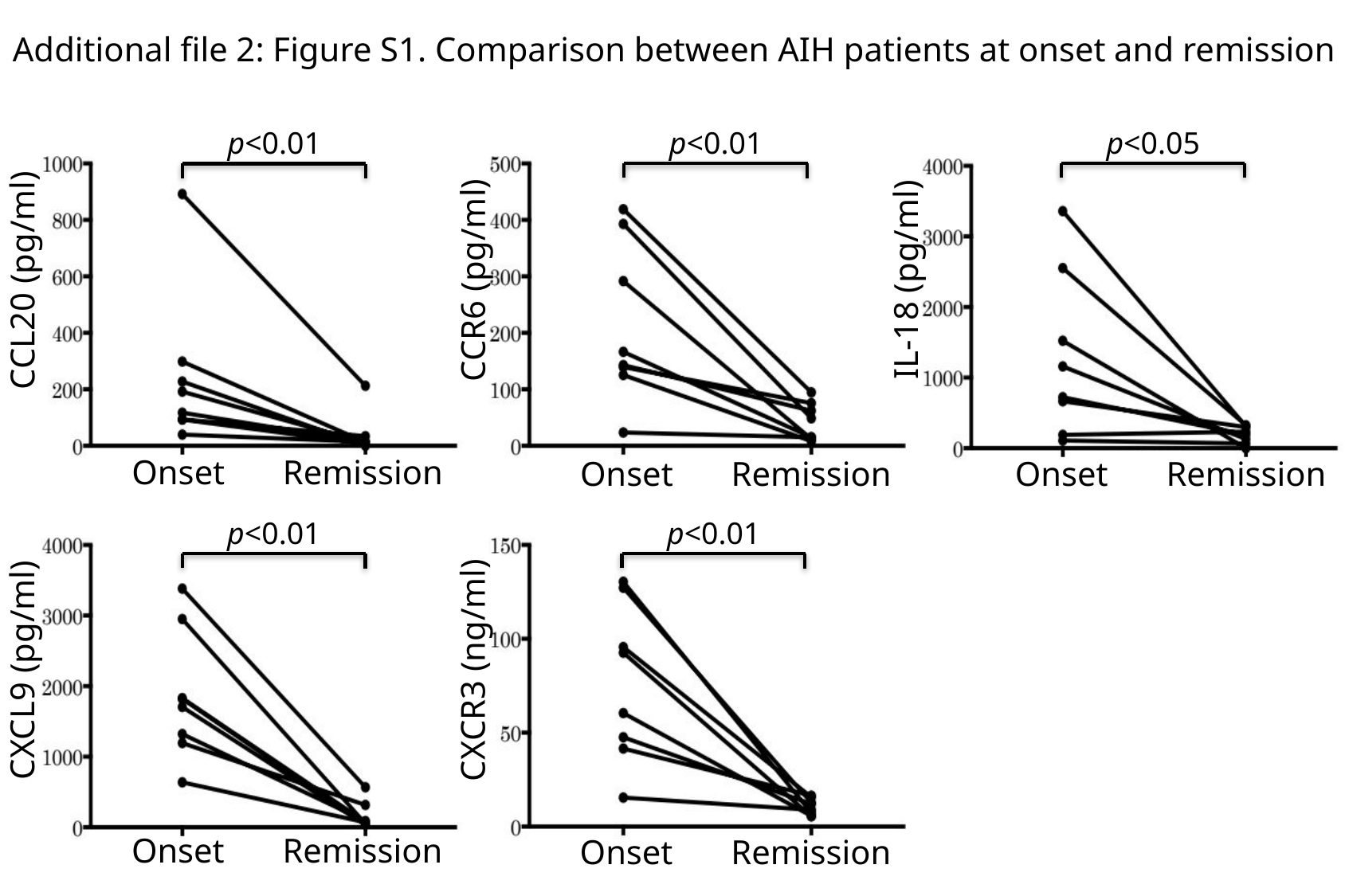

Additional file 2: Figure S1. Comparison between AIH patients at onset and remission
p<0.05
p<0.01
p<0.01
IL-18 (pg/ml)
CCR6 (pg/ml)
CCL20 (pg/ml)
Onset
Remission
Onset
Remission
Onset
Remission
p<0.01
p<0.01
CXCR3 (ng/ml)
CXCL9 (pg/ml)
Onset
Remission
Onset
Remission

## Slide 2
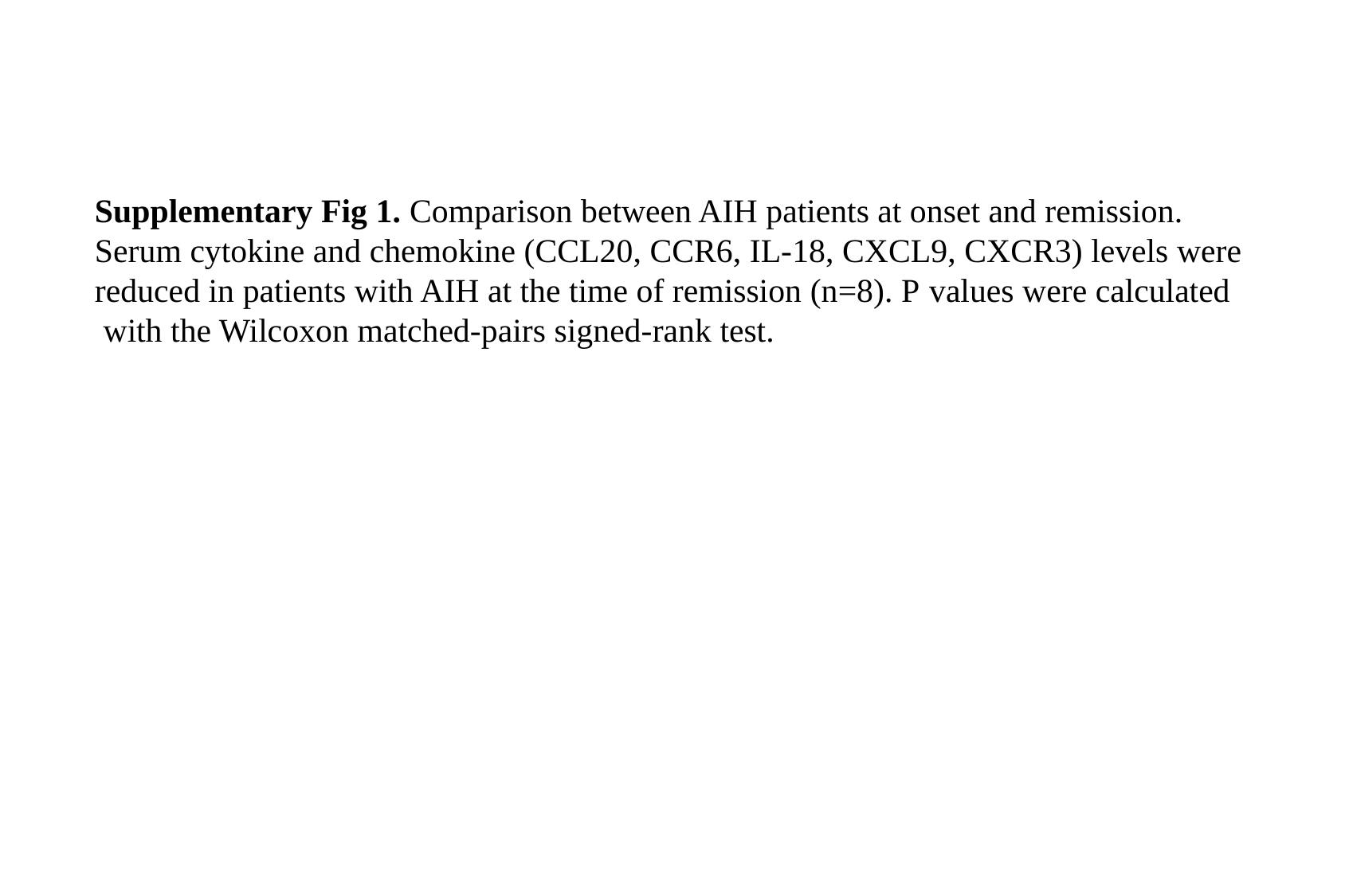

Supplementary Fig 1. Comparison between AIH patients at onset and remission.
Serum cytokine and chemokine (CCL20, CCR6, IL-18, CXCL9, CXCR3) levels were
reduced in patients with AIH at the time of remission (n=8). P values were calculated
 with the Wilcoxon matched-pairs signed-rank test.
